# A Proteomic View of Butterfly Metamorphosis

**DOI:** 10.3390/proteomes13040068

**Published:** 2025-12-18

**Authors:** Andrew Hesketh, Juned Kadiwala, Vaishnavi Ravikumar, Ana Rita Garizo, Patrícia Beldade, Marjorie Fournier, Rameen Shakur

**Affiliations:** 1 Brighton Integrative Genomics (BIG) Unit and the Centre for Precision Health and Translational Medicine, School of Applied Sciences, University of Brighton, Brighton BN2 6DN, UK; a.hesketh@brighton.ac.uk (A.H.); kadiwalajuned@gmail.com (J.K.); 2 Advanced Proteomics Facility, Department of Biochemistry, University of Oxford, Oxford OX1 3QU, UK; vaishnavi.ravikumar@bioch.ox.ac.uk (V.R.); marjorie.fournier@bioch.ox.ac.uk (M.F.); 3 Center for Ecology, Evolution and Environmental Changes (cE3c) & Global Change and Sustainability Institute (CHANGE), Faculty of Sciences, University of Lisbon (FCUL), 1749-016 Lisbon, Portugal; argarizo@ciencias.ulisboa.pt (A.R.G.); pbeldade@ciencias.ulisboa.pt (P.B.)

**Keywords:** metamorphosis, bottom-up proteomics, LFQ, insect, butterfly, *Bicyclus anynana*

## Abstract

Background: Insect metamorphosis is one of the most fascinating developmental processes in the natural world. Complete metamorphosis requires the breakdown and reorganisation of larval tissues and the coordinated construction and development of adult structures. The molecular events that achieve this transformation are, however, incompletely understood, and there is a particular shortage of data describing changes in protein abundance that occur during the process. Methods: Here, using a label-free quantitative bottom-up approach, we perform a novel whole-organism proteomic analysis of consecutive developmental stages of male *Bicyclus anynana* butterflies as they develop from caterpillars into adults via pupation. Results: Our analysis generated a dynamic reference dataset representing 2749 detected proteins. Statistical analysis identified 90 proteins changing significantly in abundance during metamorphosis, and functional interpretation highlights cuticle formation, apoptosis and autophagy during the pupal stages, and the up-regulation of respiration and energy metabolism upon completion of the fully formed adult. A preliminary search for potential peptide phosphorylation modifications identified 15 candidates, including three proteins with roles in muscle function. Conclusions: The study provides a basis for future protein-level analysis of butterfly metamorphosis and suggests the importance of dissecting the post-translational regulation associated with this fascinating developmental transformation.

## 1. Introduction

Insect metamorphosis is a complex developmental process entailing a dramatic remodelling of the body plan between life stages. The transformation of larvae specialised in feeding and growth into adults that can disperse and reproduce takes place through a non-feeding and immobile transitional pupal stage, in which tissues are extensively remodelled and rebuilt [1,2]. Metamorphosis is tightly coordinated by two main hormonal systems, ecdysteroids and juvenile hormones [3], and has been extensively studied in *Drosophila melanogaster* [4,5,6,7]. However, metamorphosis in other insects, including the Lepidoptera, which is one of the three most species-rich insect orders and the largest evolutionary radiation of herbivorous animals, remains underexplored. Furthermore, much of what we do know about the molecular orchestration of metamorphosis comes from studies at the level of gene transcription and typically focuses on specific body parts (e.g., metamorphosis of the wings, legs, or eyes) and limited developmental comparisons (e.g., larva versus pupa or larva versus adult) [8,9,10,11]. There is a notable shortage of proteomics data quantifying the global changes in protein abundance that occur across the entire metamorphic journey.

In recent work, we used Oxford Nanopore long-read sequencing to explore the changes in DNA cytosine methylation and gene transcription taking place during pupation of the laboratory butterfly *Bicyclus anynana* [12]. This identified significant changes in the expression of ~4700 genes during the developmental progression from 5th instar larvae to late-stage pupae containing fully developed adults about to eclose. Functional analysis of these changes defined a progression from lipid metabolism in larvae towards an up-regulation of muscle formation and mitochondrial energy generation in pupae. In this study we extend this analysis to quantify the changes taking place in the proteome across a comparable set of five consecutive developmental stages using a label-free quantitative bottom-up approach. We identify a subset of proteins changing significantly in abundance during metamorphosis, interpret these changes in the context of existing functional annotation, and perform a preliminary search to identify any proteins that may be modified by serine, threonine or tyrosine phosphorylation.

## 2. Materials and Methods

### 2.1. Insect Rearing and Sampling

**Figure 1:**
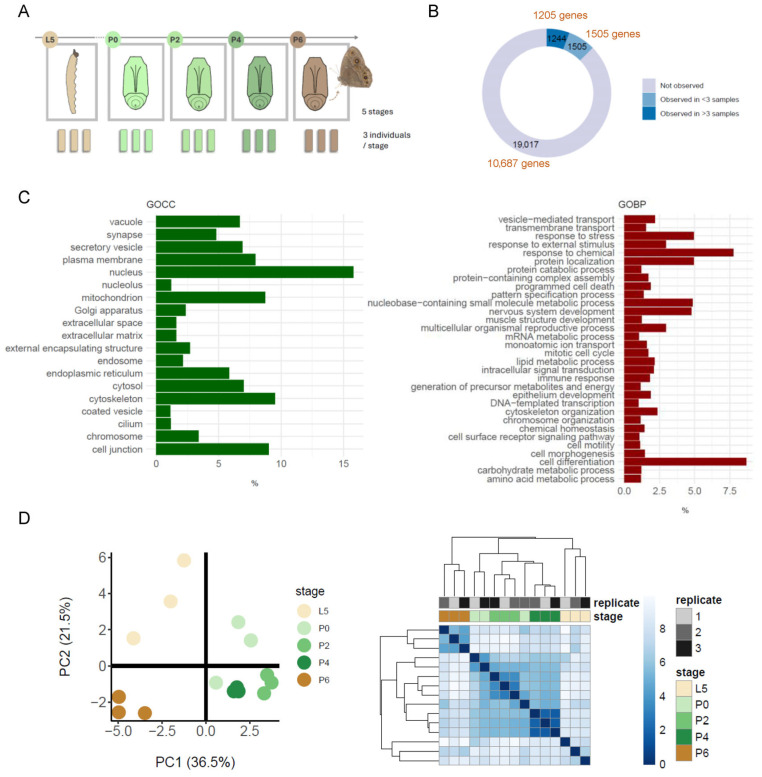
The *Bicyclus anynana* proteome during metamorphosis. (**A**) Male *B. anynana* individuals were sampled in triplicate for proteome analysis during progression from 5th and final instar larvae (L5) to the day of pupation (P0), and 2 (P2), 4 (P4) and 6 (P6) days post-pupation when fully formed butterflies are about to eclose. (**B**) The number of proteins in the annotated *B. anynana* proteome search database that were identified in the samples are grouped according to their detection in 0 (Not observed), 1–3 (Observed in <3) or more than 3 (Observed in >3) of the 15 samples analysed. The corresponding number of gene loci from which the proteins are derived are indicated in brown text. (**C**) Cellular component (GOCC) and biological process (GOBP) GO Slim mapping plots showing the diversity of functions represented by the reliably detected set of 1244 proteins from 1205 genes. Each plot shows the proportion of the gene annotations (*x*-axis) that are associated with a specific GO Slim term (*y*-axis) within each ontology subset. (**D**) Unsupervised clustering by PCA (left) and hierarchical clustering of Euclidean distances (right) showing the relationship between all 15 samples in terms of protein content.

A captive population of *Bicyclus anynana* [13] was maintained and sampled (*n* = 3 for each of 5 developmental stages) under laboratory conditions. Three biological replicates (and no technical replicates) were analysed, with the goal of generating sufficient data to begin to view the main trends in protein abundance across the five stages of metamorphosis studied within the limits of the resources available. Animals were kept at 27 °C with 65% humidity and a 12 h light/12 h dark photoperiod. About 200 male larvae housed together in the same cage were fed ad libitum with young maize plants. Specimens were then collected for subsequent protein extraction by placing them inside a microtube and flash freezing in liquid nitrogen (30 sec exposure) at the following consecutive stages of development: 5th and final instar larvae (L5); pupae on the day of pupation (P0); two days into pupation (P2); four days into pupation (P4); and six days into pupation (P6), at which point a fully formed adult butterfly is about to eclose out of the pupal case (Figure 1A).

### 2.2. Protein Extraction and Digestion

Samples were stored at −80 °C prior to tissue lysis using Eppendorf micro pestles in combination with the Kimble Pellet Pestle Motor, ensuring thorough mechanical disruption. Proteins in the lysates were precipitated using 20% trichloroacetic acid and washed twice with cold acetone. Protein pellets were air-dried before redissolving in denaturation buffer containing 8 M urea and 0.1 M ammonium bicarbonate (pH 8.0). A total of 10 µg of protein was reduced with 10 mM tris(2-carboxyethyl) phosphine for 30 min at 24 °C with shaking at 800 rpm, followed by alkylation with 50 mM 2-chloroacetamide (Sigma-Aldrich, St. Louis, MO, USA) for 30 min in the dark under the same conditions. Serial digestion was performed, first using Lys-C (FUJIFILM Wako Chemicals Europe, Neuss, Germany), at an enzyme-to-protein ratio of 1:100 *w*/*w* for 2 h at 37 °C, then with trypsin (Promega, Madison, MO, USA) at an enzyme-to-protein ratio of 1:40 *w*/*w* overnight (16–20 h) at 37 °C, both with shaking at 750 rpm. Digestion was quenched with 5% formic acid, and peptides were desalted using C18 StageTips [14]. Peptides were eluted from the tips in 50% acetonitrile/0.1% formic acid and dried in a vacuum centrifuge prior to resuspension in 5% acetonitrile/5% formic acid, ready for label-free LC-MS/MS analysis.

### 2.3. LC-MS/MS Analysis

LC-MS/MS was conducted using separation by nano liquid chromatography (Thermo Scientific Ultimate RSLC 3000, Thermo Fisher Scientific, Waltham, MA, USA) coupled to a Q Exactive mass spectrometer equipped with an Easy-Spray source (Thermo Fisher Scientific, Waltham, MA, USA). Peptides were first trapped onto a C18 PepMac100 precolumn (300 µm i.d. × 5 mm, 100 Å, Thermo Fisher Scientific, Waltham, MA, USA) using Solvent A (0.1% formic acid, HPLC grade water), then separated on an Easy-Spray RSLC C18 column (75 µm i.d., 50 cm length, Thermo Fisher Scientific, Waltham, MA, USA) using a linear gradient of 15% to 35% solvent B (0.1% formic acid in acetonitrile) over 120 min at a flow rate of 200 nL/min. Raw data were acquired in data-dependent acquisition mode. Full-scan MS spectra were acquired in the Orbitrap using the following settings: scan range 350–1500 *m*/*z*; resolution 70,000; automatic gain control (AGC) target 3 × 10^6^; and 50 ms maximum injection time. The ten most intense peaks were subjected to higher energy collision dissociation (HCD) fragmentation using 30% of normalised collision energy. HCD spectra were acquired using the following settings: resolution 17,500; AGC target 5 × 10^4^; maximum injection time 120 ms; fixed mass 180 *m*/*z*; charge exclusion for unassigned and 1+ ions; and 40 s dynamic exclusion.

### 2.4. MS/MS Data Processing

Following data acquisition, tandem mass (MS/MS) spectra were searched using Sequest HT in Proteome Discoverer software version 1.4 against a protein sequence database containing 19,579 protein entries, including 12,296 *Bicyclus anynana* (butterfly) proteins and 283 common contaminants. The database was constructed from the proteoforms annotated in the NCBI RefSeq genome GCF_947172395.1_ilBicAnyn1.1 downloaded in August 2023. The following mass adjustment settings were applied to consider potential amino acid modifications during database searching: full carbamidomethylation of cysteines (+57.0215, statically added); oxidation of methionines (+15.9949, dynamically added); acetylation of N-terminal amino acids (+42.0106, dynamically added); and phosphorylation of serine, threonine or tyrosine (+79.966, dynamically added). Up to a maximum of two missed cleavages were permitted, and peptide mass tolerance on the precursor and fragment ions was 20 ppm and 0.6 Da, respectively. Data were filtered at the peptide-spectrum match level, applying an FDR threshold of ≤1%. Putative phosphopeptides were initially identified from their mass changes and manually validated by visual inspection of the MS/MS spectra.

### 2.5. Downstream Processing and Analysis of Protein Abundance Data

Normalised spectral abundance factors (NSAF) for all the detected proteins were filtered to remove those proteins with zero NSAF values for more than 12/15 of the samples (1244 proteins), then analysed for differential abundance between all pairs of stages using the power law global error method (PLGEM) using the run.plgem function in the R package plgem with default settings [15] and requiring a false positive rate *p* ≤ 0.001 for significance. When testing 1244 proteins, an average of 1.2 proteins can be expected to be identified by chance using this threshold. Zero NSAF values were treated as zero (i.e., below the limit of detection) and not subjected to imputation. Any protein identified as being significantly different in abundance between any of the ten possible inter-stage comparisons was defined as being differentially abundant during the developmental time course. Unsupervised clustering was performed in R using principal components analysis (PCA) and hierarchical clustering based on Euclidean distances. GO Slim mappings were performed using the R package GSEABase (version 1.70.0) and the goslim_drosophila.obo annotation [16]. The R package clusterProfiler [17] was used for functional over-representation analysis of groups of significant genes of interest, using Gene Ontology and KEGG annotations previously produced for *B. anynana* [12], and applying a significance threshold of 5% to the Benjamini and Hochberg corrected *p*-values to select significantly over-represented functional categories. The genes encoding the total *B. anynana* proteome were used as background sets for the over-representation analyses. Hierarchical clustering of data was performed in R using the pheatmap package, scaling the data to produce row-wise z-scores before clustering [18]. For comparisons with gene transcription, DESeq2 normalised expression values and log2 fold-change values for changes in expression between developmental stages were obtained from Hesketh et al. [12]. The mean of the triplicate gene transcript and protein values were analysed in the correlations.

## 3. Results and Discussion

### 3.1. The Observable Proteome During B. anynana Metamorphosis

In this study we quantified changes in the proteome of an experimental model butterfly, the species *B. anynana* [13], in triplicate samples derived from the whole organism and covering the entire metamorphosis process, the larva to pupa transition as well as the progression through pupal development until a fully developed adult is about to eclose out of the pupal case (Figure 1A). A total of 2749 proteins (from 2710 genes) were quantified using a label-free approach in a bottom-up LC-MS/MS analysis of the five consecutive developmental stages sampled, with 1244 being defined as reliably quantified due to their inclusion in three or more of the 15 samples analysed (Supplementary Data S1). At the gene level, this corresponds to a dataset describing the quantification of 1244 protein products from 1205 gene loci, representing expression from 8.8% of the genes in the genome (Figure 1B). The 1244 proteins from these 1205 genes are the subject of all downstream analyses reported here. The genes represent a diverse range of cellular locations and functions as determined by GO Slim mapping using the *Drosophila melanogaster* Slim categories from the Gene Ontology Consortium [19,20] (Figure 1C). While these results come with the caveat that they are based on *D. melanogaster* GO Slim annotations, no GO Slim categories are currently available for *B. anynana,* and the use of those available from the closest and most biologically relevant species offers a useful approach to illustrate the broad themes in the *B. anynana* proteins detected. GO Slim mapping based on the Cellular Component ontology (GO CC) indicates proteins associated with the plasma membrane, Golgi apparatus and endoplasmic reticulum, as well as cytosolic, nuclear, mitochondrial, cytoskeletal, vacuolar, and extracellular proteins. Similar analysis using the Biological Process ontology indicates the representation of proteins with roles in a wide variety of processes. These include cell-level processes, such as cell division, transcriptional regulation, cell metabolism, programmed cell death, and cell differentiation, as well as higher-level processes, such as transport processes, nerve and muscle development, immune response, and response to stimuli and signals.

The bottom-up shotgun proteomics approach that we used is an efficient and sensitive method for the high-throughput identification and quantification of proteins in the complex whole organism samples in the experiment, but it comes with limitations. Since the analysis is based on peptide fragments, and a single peptide can potentially be assigned to multiple different proteoforms, it generates data that is unable to accurately capture and quantify the full complexity of the proteome. In addition, quantification based on label-free LC-MS/MS is less reproducible compared to label-based proteomics methods and is more prone to producing data with missing values across the different samples. Highly abundant proteins can dominate the spectra, masking signals from low-abundance ones. We also note that the protein precipitation step that was used during sample preparation is likely to exclude low molecular weight proteins (<15–20 kDa) from the data.

### 3.2. Differential Protein Abundance During Metamorphosis

**Figure 2:**
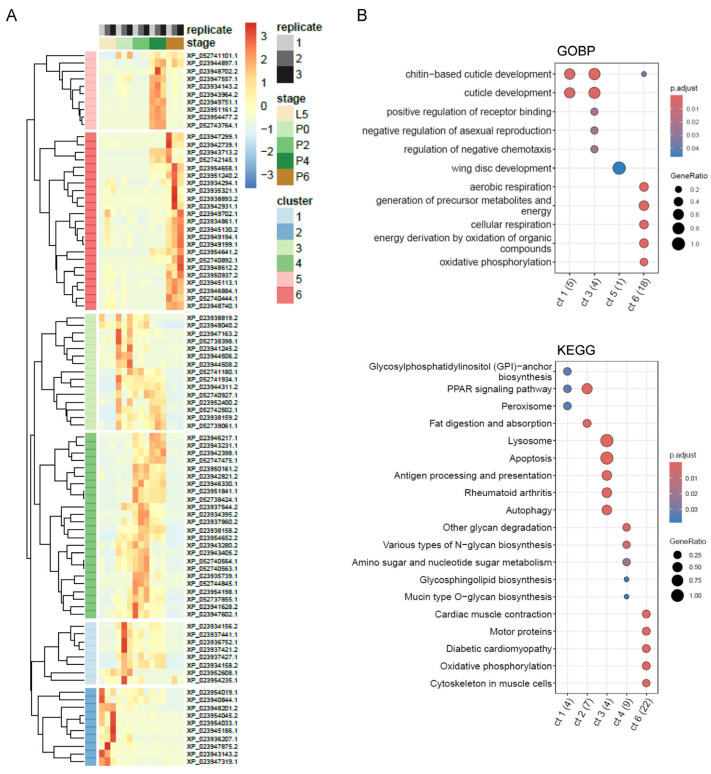
Significant changes in protein abundance during metamorphosis. (**A**) Hierarchical clustering of the normalised abundance data for the 90 significantly differently abundant proteins defines six clusters characterising the major changes in protein abundance during progression from L5 to P6. Protein accession numbers are shown for each row, and cluster membership is listed in Supplementary Data S2. Heatmap colours correspond to the row-wise z-scores of the NSAF values. All three biological replicates are shown. (**B**) Functional analysis of each co-abundance cluster using Gene Ontology Biological Process (GOBP) and KEGG annotations previously produced for *B. anynana* [12], highlighting significant over-representation (*padj* ≤ 0.05) of functional categories in specific clusters. Clusters are indicated on the *x*-axis, and the numbers shown in parentheses indicate the number in each cluster that possessed an annotation (GOBP or KEGG). Only up to five of the most significant categories for each cluster are plotted here; full results are presented in Supplementary Data S4.

Unsupervised clustering of the normalised abundance data for the set of 1244 proteins using PCA and hierarchical clustering based on Euclidean distances clearly distinguished the L5 larval samples from the intermediate P0, P2 and P4 pupal stage samples and from the mature P6 adult pupae (Figure 1D). The expectation that samples replicated from the same developmental stage should cluster more closely with each other than with samples from different stages is met, although there is some spreading for the data for the L5 and P0 replicates. This suggests the presence of some noise in the data for these samples, which may be biological or technical in origin. Statistical analysis identified 90 proteins showing significant differential abundance between developmental stages (*p* ≤ 0.001) (Supplementary Data S2). Hierarchical clustering of these differentially abundant proteins grouped the data into six profiles defining the coordinated changes in protein abundance taking place during metamorphosis (Figure 2A, Supplementary Data S3). Functional over-representation analysis of the clusters identified indicates an up-regulation in proteins with functions associated with respiration, energy generation and muscles in the mature adult pupal stage P6 (cluster 6 in Figure 2A) (Figure 2B). This is consistent with previous observations obtained from an analysis of the transcriptome under directly comparable conditions [12], and it is tempting to consider that these might relate to the change in energy requirements that will accompany adult life-specific functions such as flight and mating. Similar changes in energy generation and metabolism, but also in innate immune processes, were also identified in a proteomic analysis of the larval-adult transition (via one pupal sample) in the Diamondback moth [21]. Proteins with roles in the development of the cuticle are significantly enriched in the abundance clusters showing up-regulation during both the L5 larval stage and the P2 pupal stage (clusters 1 and 3 in Figure 2A) (Figure 2B), presumably reflecting the importance of cuticle formation in both the development of the pupal case and the production of the exoskeleton for the adult body tissues (head, thorax, abdomen, legs, wings) that develop during pupation. Wing disc development is associated with the group of proteins most highly expressed in the P4 pupal stage (cluster 4 in Figure 2A), and functions associated with apoptosis and autophagy are enriched in cluster 3, which is characterised by increased abundance during the P0-P4 developmental stages (Figure 2). Table 1 highlights a selection of differentially abundant proteins identified in Figure 2A with putative functions unrelated to structural organisation (chitin, muscle) or energy generation and includes those with putative roles in fertility, perception of the environment, stress response, macromolecular degradation, pigmentation and neurone development. The identification of a bilin-binding protein is potentially interesting in the context of pupal colour polyphenism. Changes in the abundance of bilin-binding proteins have been identified in a combined proteome and transcriptome study of the polyphenism of larval-pupal pigmentation in *Papilio xuthus* butterflies [22].

The relatively low proportion of proteins identified as being differentially abundant during the experiment is likely to be the result of the small sample size (*n* = 3 for each developmental stage) and the known issues associated with the reproducibility of label-free quantification discussed above. Studies focusing on specific aspects of Lepidopteran metamorphosis using iTRAQ-based quantitative proteomics report the identification of larger numbers of differentially abundant proteins [21,22,23]. It should also be noted that to minimise variability in the current experiment and to generate data directly comparable to our recent transcriptomic and epigenomic analysis of metamorphosis [12], only male butterflies were studied, and information on any female-specific developmental processes will consequently be absent from the data collected. Future interpretation of the developmental changes in protein abundance taking place would be strengthened by collecting detailed observations of the biological changes taking place in the organism at the time of sampling and by increasing the sample size to improve statistical power. Furthermore, while the analysis of samples derived from the whole organism generates an unbiased overview of the changes in protein abundance occurring, it comes with the limitation of generating data that is an average across all tissues present and masks tissue-specific programmes. Future application of spatial proteomics approaches will be important for accessing these localised developmental changes.

### 3.3. The Relationship Between Gene Transcription and Gene Product Abundance, and a Preliminary Search for Phosphopeptides

**Figure 3:**
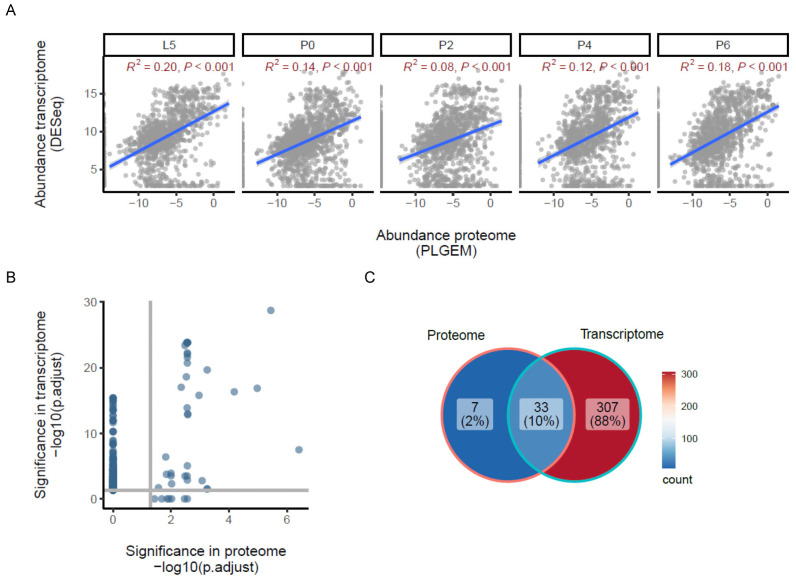
Correlation between changes in transcription and protein abundance. (**A**) Linear regression analysis showing the relationship between the proteome (*x*-axis) and transcriptome (*y*-axis) data for each developmental stage. All abundances are the mean of log2 normalised values for each stage (*n* = 3), and significant R^2^ values are shown (*p* ≤ 0.001). (**B**) Significant GOBP functional categories represented in the proteins up-regulated in abundance in the P6 stage (cluster 6 in Figure 2A) (*x*-axis) compared with significant GOBP functional categories represented in the transcripts up-regulated in abundance in the P6 stage (cluster 6 in Figure 1 from Ref. [12]) (*y*-axis). Both axes show the −log10 (p.adjust) such that more significant results are displayed at higher values, and each point plotted represents a different GOBP category that is significant in at least one of the datasets (p.adjust ≤ 0.05, marked on each axis by horizontal and vertical lines). (**C**) Overlap between the significant GOBP functional categories considered in panel B. The threshold for significance in each case was p.adjust ≤ 0.05.

A comparison of the proteomics data in this study with transcriptome data we have previously generated for the same developmental time course (but sampling different individuals) [12] provided the opportunity to visualise the relationship between the transcriptome and proteome during metamorphosis (Figure 3). This was performed at the level of canonical proteins, not proteoforms, due to the technical limitations of the quantification by shotgun proteomics previously discussed. Linear regression identified statistically significant but weak correlations between the protein and mRNA abundances for the data from all five stages, with the strongest correlations being observed for L5 and P6 (R^2^ = 0.20 and 0.18, respectively; Figure 3A). Comparing the fold changes detected between the pairs of developmental stages using each platform, an approach that minimises the influence of the differences in data normalisation, also gave similar results (Supplementary Figure S1). While the most accurate comparisons to make would be between individual mRNA transcript splice variants and their corresponding proteoforms, this gene-level analysis indicates an overall relationship between mRNA levels and protein abundances at the global level but also suggests that other factors likely play a substantial role in determining the abundance of individual proteins. These post-transcriptional mechanisms are likely to include the regulation of translation efficiency and differences in the relative stabilities of the mRNA and protein species for each gene product [24,25]. These processes are known to play important roles in metamorphosis, where high turnover of protein is required for the extensive reorganisation of tissues during the pupal stage [2,26], and developmental regulation by microRNAs and mRNA modifications dynamically controls post-transcriptional mRNA transcript stability [27,28,29,30]. The weaker correlations observed for the P0 to P4 pupal samples compared to the L5 larval and P6 adult stages are consistent with this. At the level of functional significance, a comparison of the Gene Ontology Biological Process (GO BP) categories identified as being significant in the group of proteins identified as being up-regulated in abundance in the proteome data in the mature pupal stage P6 (cluster 6 in Figure 2A) with those derived from the corresponding cluster of transcripts similarly up-regulated at stage P6 in the transcriptome data shows a high level of agreement (Figure 3B). Of the 40 significant GO BP categories identified in the proteomics data, 37 were also identified as significant in the analysis of the transcriptome (Figure 3C). These categories are predominantly associated with active cellular metabolism (respiration, mitochondrial function, tricarboxylic acid cycle, purine nucleotide biosynthesis and amino acid biosynthesis).

In addition to providing quantitative data on protein abundance, LC-MS proteomics offers the opportunity to qualitatively identify possible post-translational modifications (PTMs) that may regulate protein activity or localisation. We searched the peptide mass spectrometry data for preliminary evidence of phosphorylation on serine, threonine or tyrosine residues and identified 16 possible phosphopeptides, belonging to 15 different parent proteins (Table 2 and Supplementary Figure S2). Although our study is not specifically designed to quantify phosphopeptides, protein phosphorylation is central to the functioning of signal transduction pathways in eukaryotic organisms [31], and there is a lack of data describing protein phosphorylation during butterfly metamorphosis. Six of the putative phosphopeptides identified were detected in more than one sample, and visualisation of their abundance relative to that obtained for the total protein (modified and unmodified) is suggestive of a stage-dependent phosphorylation for three proteins with muscle-related functions (XP_023950391, myosin regulatory light chain 2; XP_052741313, myosin heavy chain; XP_052738629, PDZ and LIM domain protein Zasp) (Supplementary Figure S3). Although preliminary, these data, supported by previous observations describing changes in the phosphoproteome of *Helicoverpa armigera* during larval-pupal metamorphosis [32], suggest that a future dedicated phosphoproteomics study using a phosphopeptide enrichment approach will provide an important additional layer of information describing the processes important for metamorphosis.

## 4. Conclusions

In summary, we have generated quantitative mass spectrometry proteomic profiling data describing dynamic changes in protein abundance covering five consecutive stages of development during the metamorphosis of male *B. anynana* butterflies. There is a lack of data describing changes in protein abundance that occur during butterfly metamorphosis, and our analysis of samples derived from the whole organism yielded 2749 proteins, including 90 that show developmental stage-dependent changes in abundance and 15 that are likely to be phosphoproteins. The data complements previously published measurements of the transcriptome and DNA methylome during a similar developmental time course [12], and while comparisons verify a consistent identification of differentially regulated biological processes during metamorphosis between the proteomics and transcriptomics data, they indicate a weak overall correlation between the transcript and protein abundances at the individual gene level. This points to the importance of post-translational regulation during metamorphosis, which is further supported by the identification of protein candidates showing potential phosphorylation events. The study provides a basis and resource for further protein-level analysis of butterfly metamorphosis aimed at dissecting the post-translational regulation associated with this fascinating developmental transformation and how this is affected by the hormonal systems that regulate developmental transitions in holometabolous insects [3]. Given the range and diversity of the mRNA splice variants observed in the transcriptome during metamorphosis [12], the application of top-down approaches to quantify proteoforms will also have a major part to play in increasing our future understanding of metamorphosis. In the future, it will be particularly interesting to explore how the proteome of *B. anynana* is affected by temperature, as this species is a well-established model of adaptive developmental plasticity [33], where the temperature during metamorphosis affects developmental rates and trajectories to produce adult phenotypes suited to life under distinct seasonal conditions [13].

## Figures and Tables

**Table 1:** A selection of differentially abundant proteins identified in Figure 2 and their putative functions. The abundance profile cluster that each protein belongs to in Figure 2A is indicated in the Cluster column (all other proteins in Figure 2A are detailed in Supplementary Data S2).

Accession	Cluster	Product	Putative Function
XP_052741101.1	5	calphotin	light sensing
XP_023942739.1	6	delta-1-pyrroline-5-carboxylate dehydrogenase, mitochondrial	stress, metabolism
XP_023946884.1	6	calcium-transporting ATPase sarcoplasmic/endoplasmic reticulum type isoform X2	muscle contraction
XP_023938819.2	3	bilin-binding protein	pigmentation
XP_023948040.2	3	pro-cathepsin H	protease
XP_023941245.2	3	general odorant-binding protein 66-like	odour sensing
XP_052741934.1	3	testis-specific gene A8 protein-like	male fertility
XP_023943231.1	4	mesencephalic astrocyte-derived neurotrophic factor homologue	neurone development
XP_023942398.1	4	alpha-tocopherol transfer protein-like	Vitamin E
XP_023950161.2	4	apolipoprotein D	lipid metabolism
XP_023942821.2	4	fibrous sheath CABYR-binding protein	male fertility
XP_023937544.2	4	lysozyme	protease
XP_023943280.2	4	chitooligosaccharidolytic beta-N-acetylglucosaminidase	chitin degradation
XP_023943405.2	4	chitinase A	chitin degradation
XP_052740564.1	4	angiotensin-converting enzyme-like isoform X3	
XP_023954198.1	4	general odorant-binding protein 56a	odour sensing
XP_052737855.1	4	superoxide dismutase [Cu-Zn]	response to stress
XP_023948201.2	2	fatty acid-binding protein 2-like	lipid metabolism
XP_023936207.1	2	17-beta-hydroxysteroid dehydrogenase 14-like	steroid hormone biosynthesis

**Table 2:** Peptide sequences exhibiting modifications consistent with serine, threonine or tyrosine phosphorylation. Putatively phosphorylated residues are shown underlined in bold in the peptide sequence. MS/MS spectra are shown in Supplementary Figure S2.

Peptide Sequence	Protein Accession	Description	Modifications	No. Samples Identified (Max. 15)
RL**S**LNPEAGDQQAAEQNYNNYQQPEQQVK	XP_023943385.2	endocuticle structural glycoprotein ABD-4	S3(Phospho)	1
SLD**Y**IAAHPPVVEK	XP_052741399.1	larval cuticle protein LCP-17-like isoform X2	Y4(Phospho)	2
ELELQVV**T**GEVR	XP_023943025.1	rho guanine nucleotide exchange factor 7 isoform X2	N-Term(Acetyl); T8(Phospho); R12(Methyl)	6
DLILAD**Y**GKK	XP_023948915.1	pre-mRNA-processing-splicing factor 8	Y7(Phospho); K9(Methyl)	4
**T**GSNVFSMFSQK	XP_023950391.1	myosin regulatory light chain 2	T1(Phospho); M8(Oxidation)	3
GV**S**PAASIK	XP_052741313.1	myosin heavy chain, muscle isoform X31	S3(Phospho)	4
GSLADAL**T**IAPDRPYSPLAFHIPNQSLSSTSTSETQSISADK	XP_052738629.1	PDZ and LIM domain protein Zasp	T8(Phospho)	2
APQRDEDD**Y**EDVDDVPR	XP_052741581.1	uncharacterised protein LOC112049861	Y9(Phospho)	1
RTG**S**NVFSMFSQK	XP_023950391.1	myosin regulatory light chain 2	S4(Phospho)	1
IVGGTTV**S**INTYPF**S**AVLLITSGVMNR	XP_023947660.1	trypsin, alkaline B-like	S8(Phospho); S15(Phospho)	1
A**S**SLPDIYR	XP_052742507.1	monocarboxylate transporter 14	S2(Phospho)	1
RPS**S**ELVDLESFK	XP_023941594.1	uncharacterised protein LOC112048334 isoform X1	S4(Phospho)	1
AVP**T**VIS**S**EYLNTLGTK	XP_052744972.1	protein O-GlcNAcase isoform X2	N-Term(Acetyl); T4(Phospho); S8(Phospho)	1
SEK**S**LSLDLMADK	XP_023948060.2	uncharacterised protein LOC112053033	S4(Phospho); M10(Oxidation)	1
TWHI**Y**IAPLIEIFNGTSFIAMR	XP_052739755.1	solute carrier family 46 member 3	Y5(Phospho); M21(Oxidation); R22(Methyl)	1
QP**T**PHLQPMPNRLAR	XP_052739074.1	kinesin-like protein Klp10A isoform X2	N-Term(Acetyl); T3(Phospho); M9(Oxidation); R12(Methyl)	1

## Data Availability

The proteomics data generated in this study have been submitted to the ProteomeXchange Consortium via the PRIDE [34] partner repository with the dataset identifier PXD065563.

## Supplementary Materials

The following supporting information can be downloaded at https://www.mdpi.com/article/10.3390/proteomes13040068/s1: Figure S1: Correlation between changes in transcription and protein abundance; Figure S2: MS/MS spectra of the phosphorylated peptides listed in Table 2; Figure S3: Comparison of the abundances of six putative phosphopeptides from Table 2 for each developmental stage relative to the total abundance values for each protein; Data S1: Normalised Spectral Abundance Factor (NSAF) data; Data S2: Differential abundance analysis results comparing pairs of developmental stages; Data S3: The 90 proteins significantly changing in abundance during metamorphosis; Data S4: Functional enrichment analysis results for the groups of proteins in the abundance clusters shown in the heatmap in Figure 1; Data S5: Identification of peptide sequences exhibiting modifications consistent with serine, threonine or tyrosine phosphorylation.

## Abbreviations

The following abbreviations are used in this manuscript:

HCD	higher energy collision dissociation
NSAF	normalised spectral abundance factors
PLGEM	power law global error method
GO BP	Gene Ontology Biological Process
L5	5th and final instar larva
P0	pupa on the day of pupation
P2	pupa two days into pupation
P4	pupa four days into pupation
P6	pupa six days into pupation
