# Supplementary material for: A Proteomic View of Butterfly Metamorphosis
Source: Proteomes. 2025 Dec 18;13(4):68. doi: 10.3390/proteomes13040068 (PMC12737199; doi:10.3390/proteomes13040068)
Supplement: Supplementary file 1 [file proteomes-13-00068-s001.zip › Supplementary_Figures.pptx]

## Slide 1
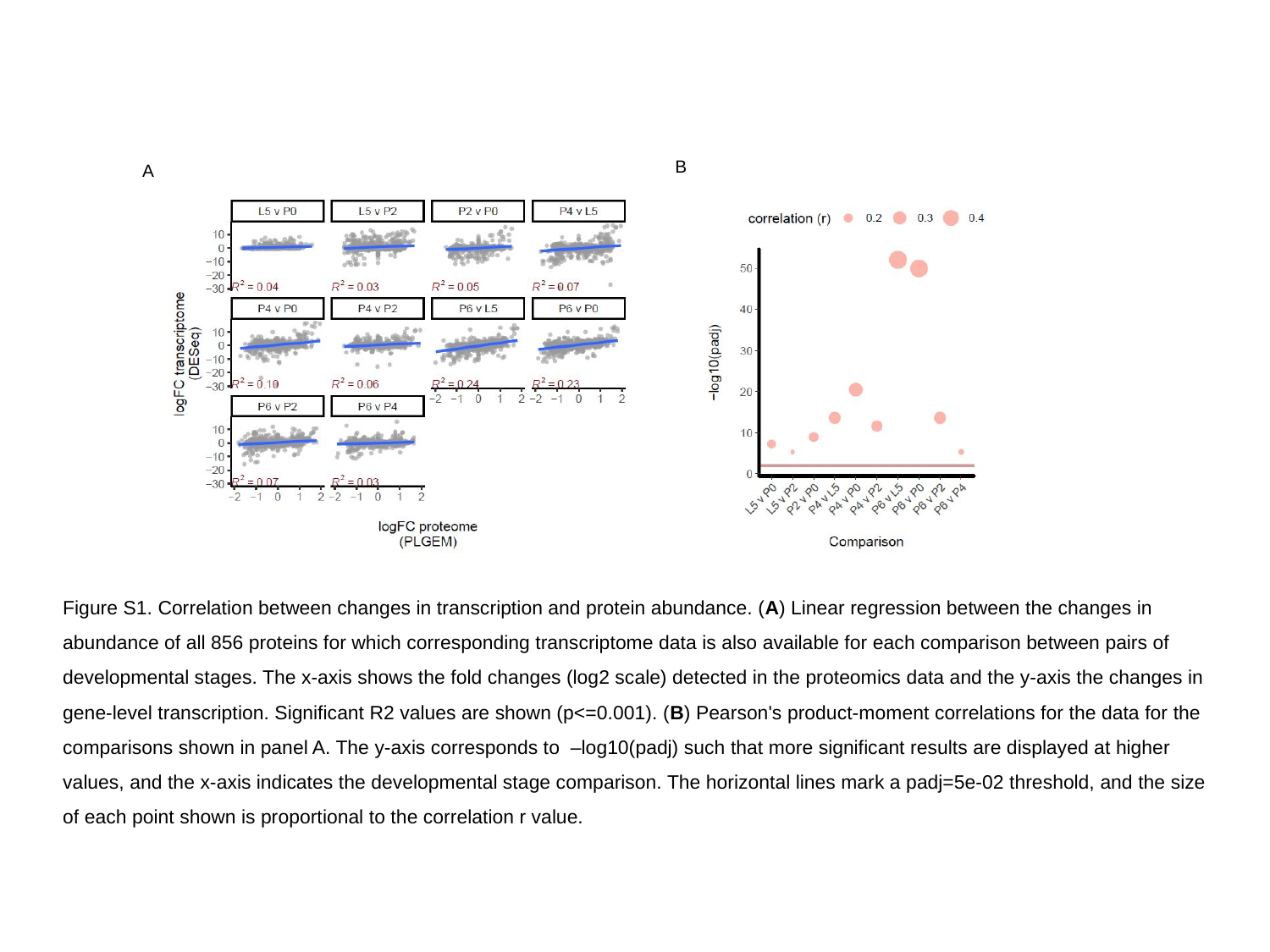

B
A
Figure S1. Correlation between changes in transcription and protein abundance. (A) Linear regression between the changes in abundance of all 856 proteins for which corresponding transcriptome data is also available for each comparison between pairs of developmental stages. The x-axis shows the fold changes (log2 scale) detected in the proteomics data and the y-axis the changes in gene-level transcription. Significant R2 values are shown (p<=0.001). (B) Pearson's product-moment correlations for the data for the comparisons shown in panel A. The y-axis corresponds to –log10(padj) such that more significant results are displayed at higher values, and the x-axis indicates the developmental stage comparison. The horizontal lines mark a padj=5e-02 threshold, and the size of each point shown is proportional to the correlation r value.

## Slide 2
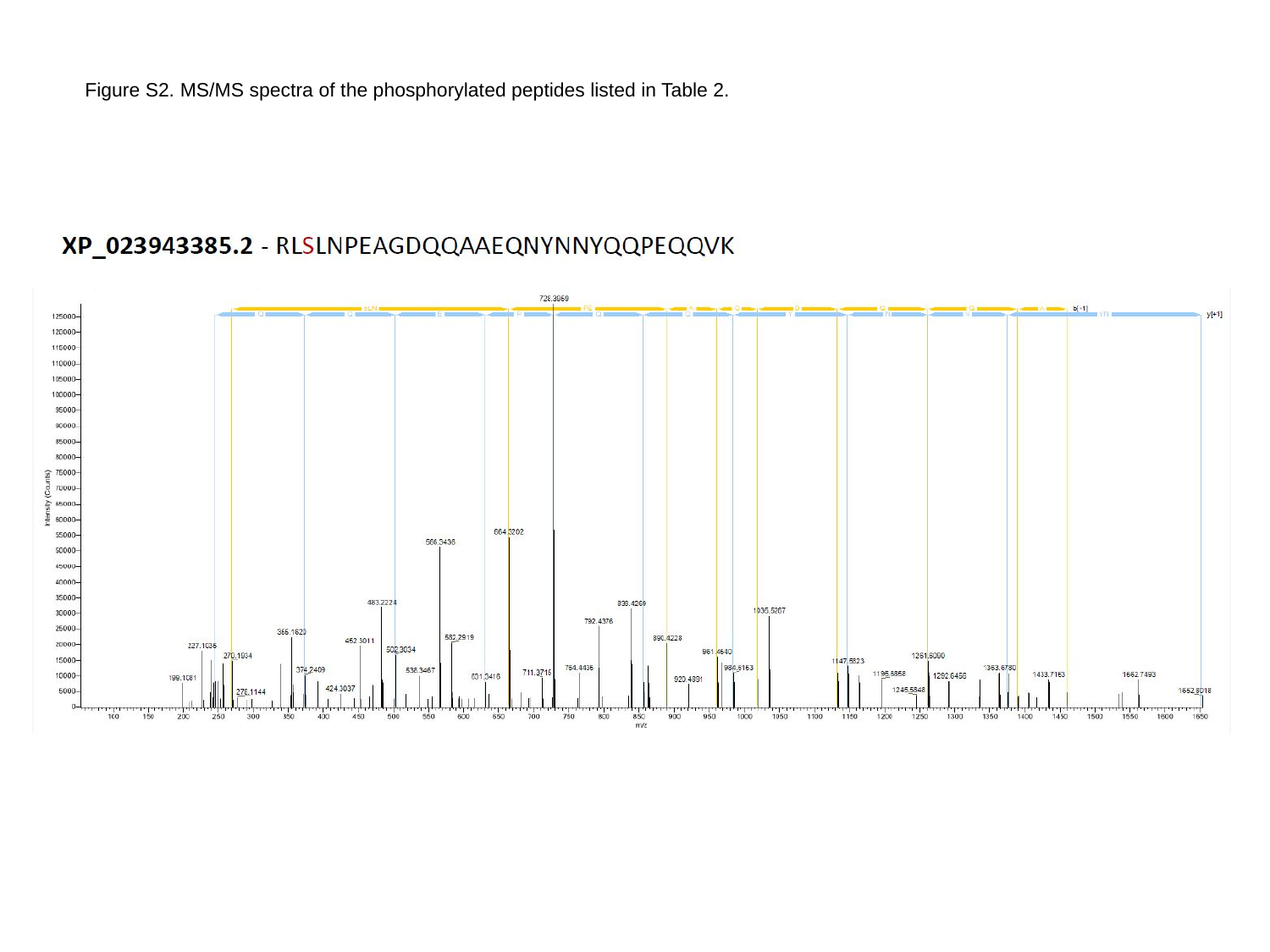

Figure S2. MS/MS spectra of the phosphorylated peptides listed in Table 2.

## Slide 3
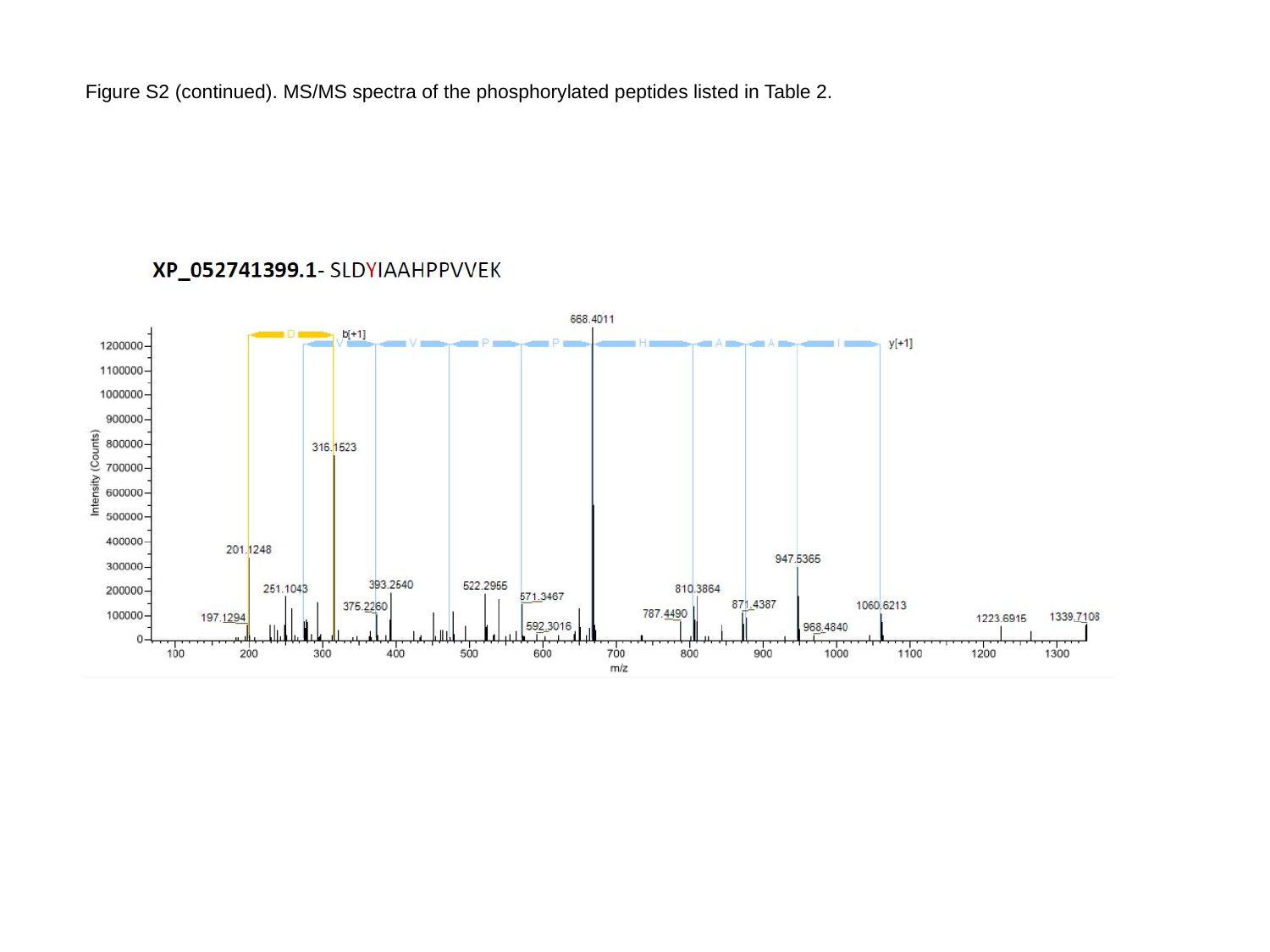

Figure S2 (continued). MS/MS spectra of the phosphorylated peptides listed in Table 2.

## Slide 4
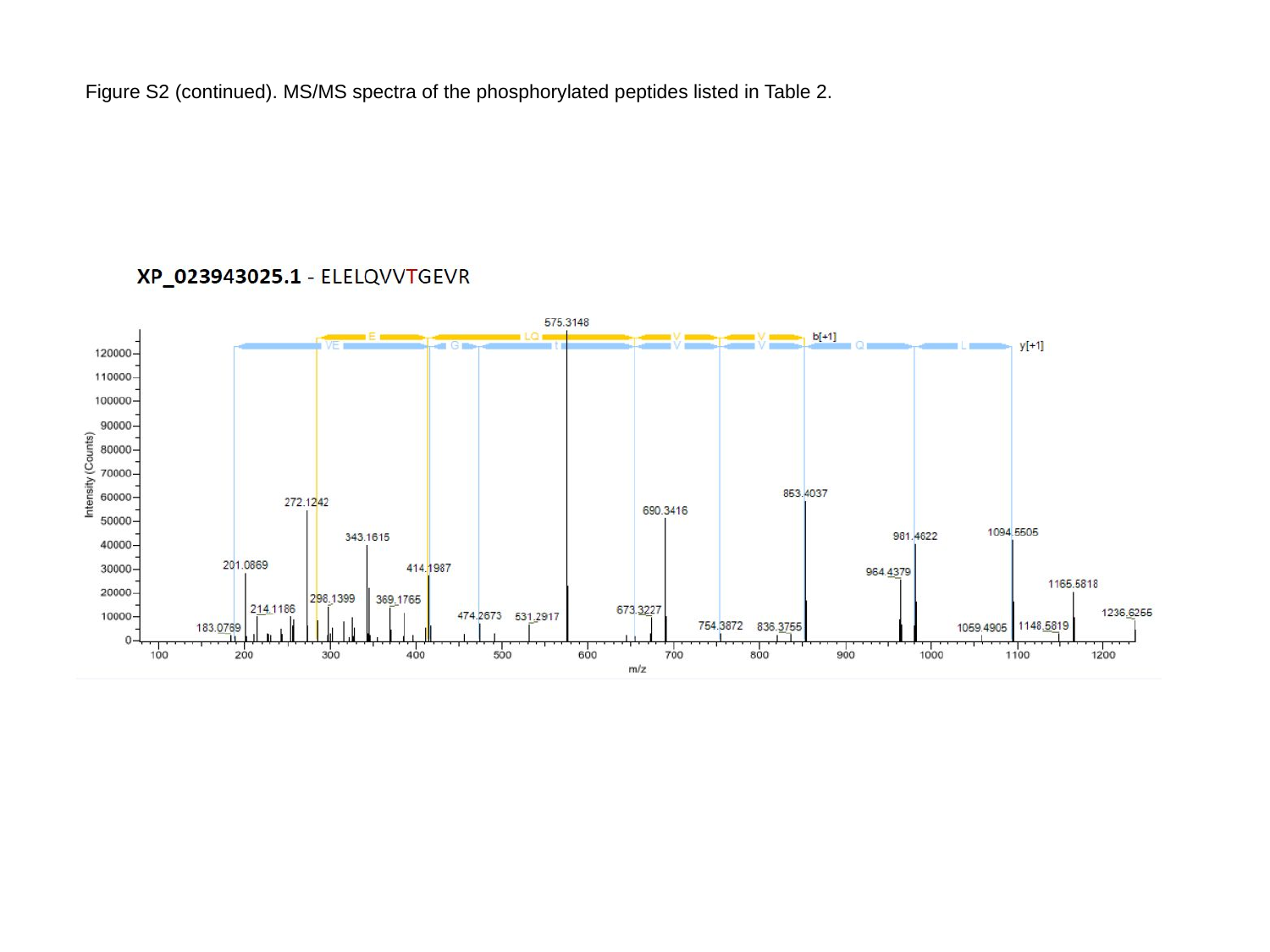

Figure S2 (continued). MS/MS spectra of the phosphorylated peptides listed in Table 2.

## Slide 5
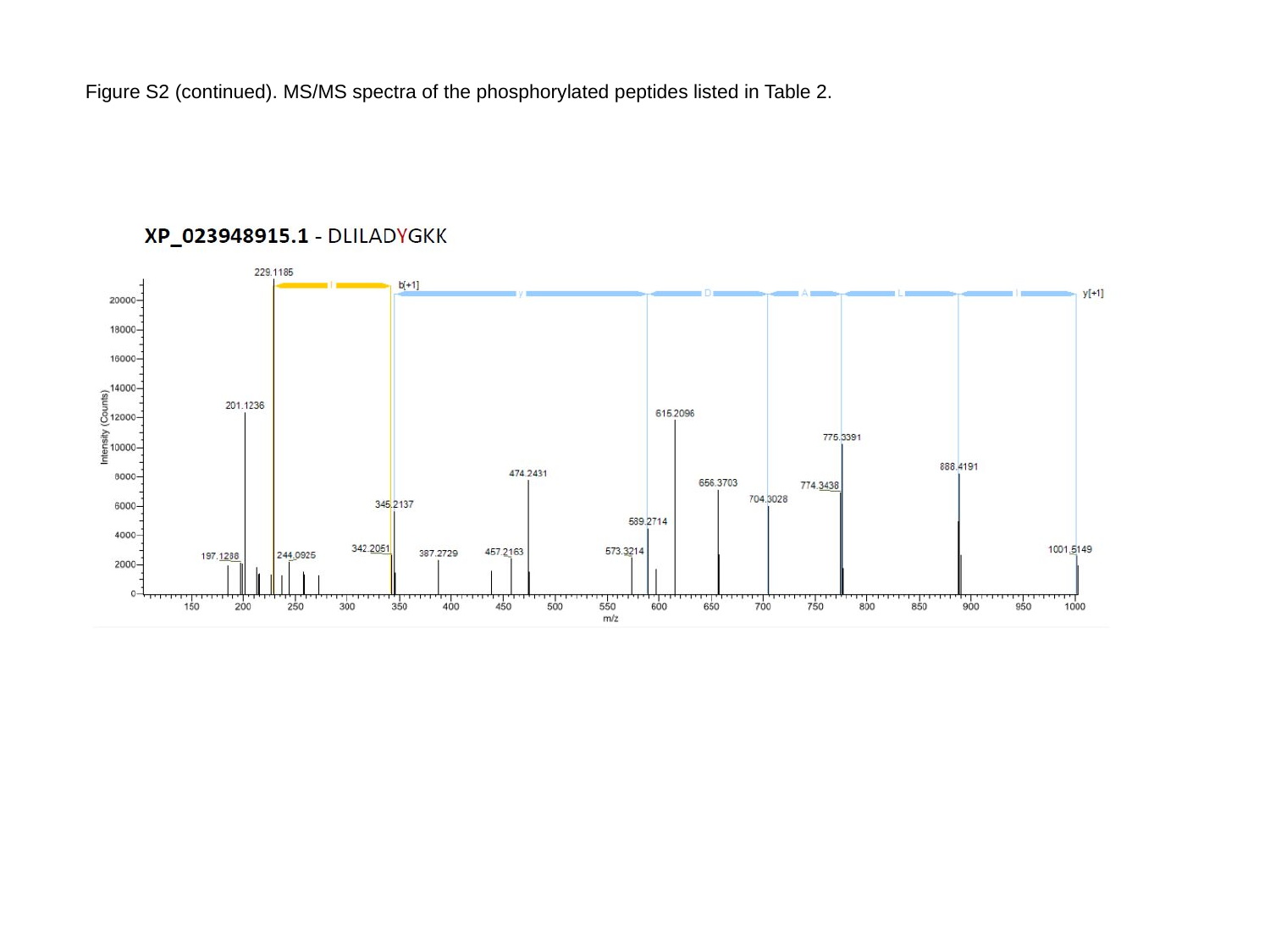

Figure S2 (continued). MS/MS spectra of the phosphorylated peptides listed in Table 2.

## Slide 6
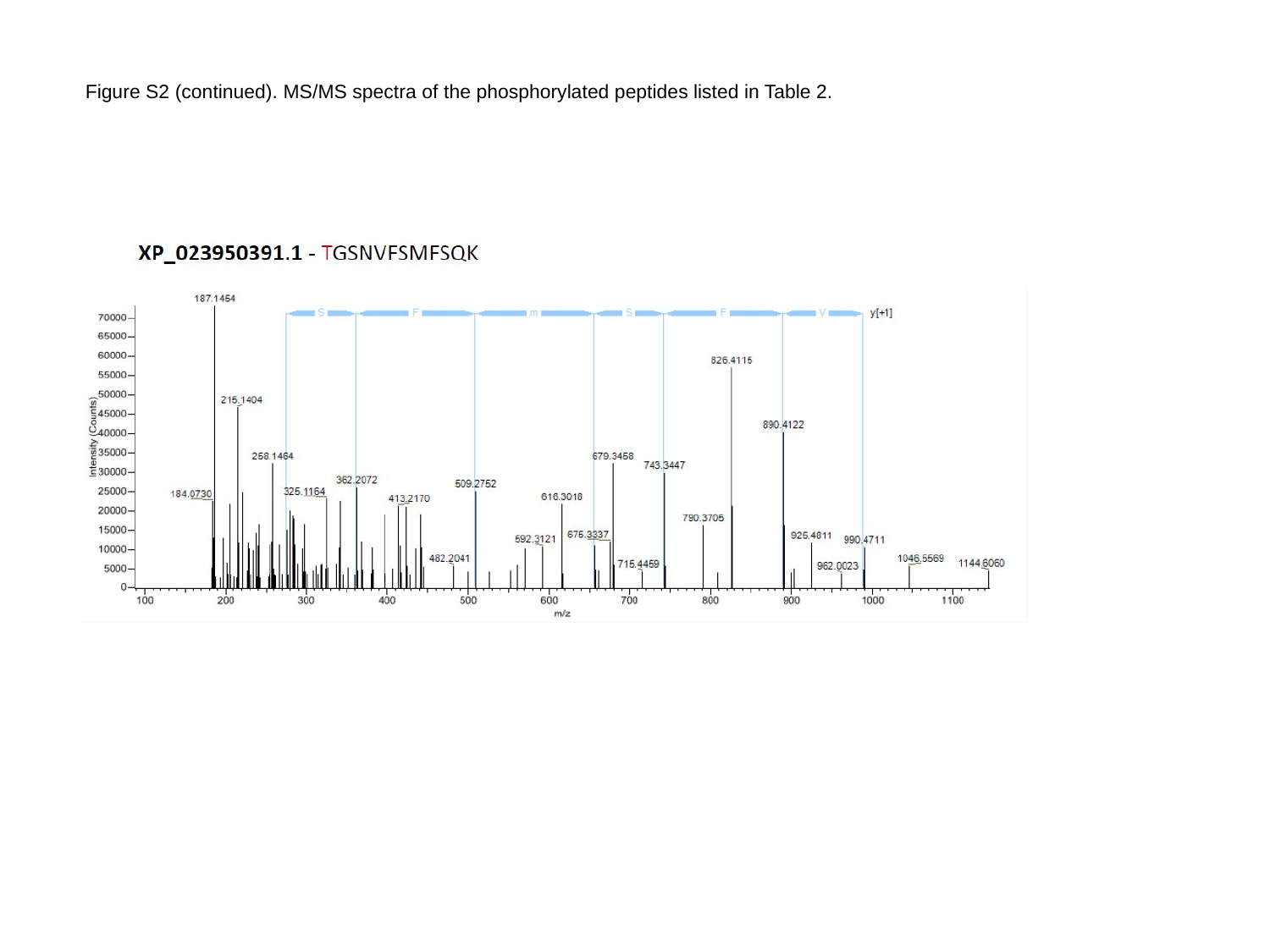

Figure S2 (continued). MS/MS spectra of the phosphorylated peptides listed in Table 2.

## Slide 7
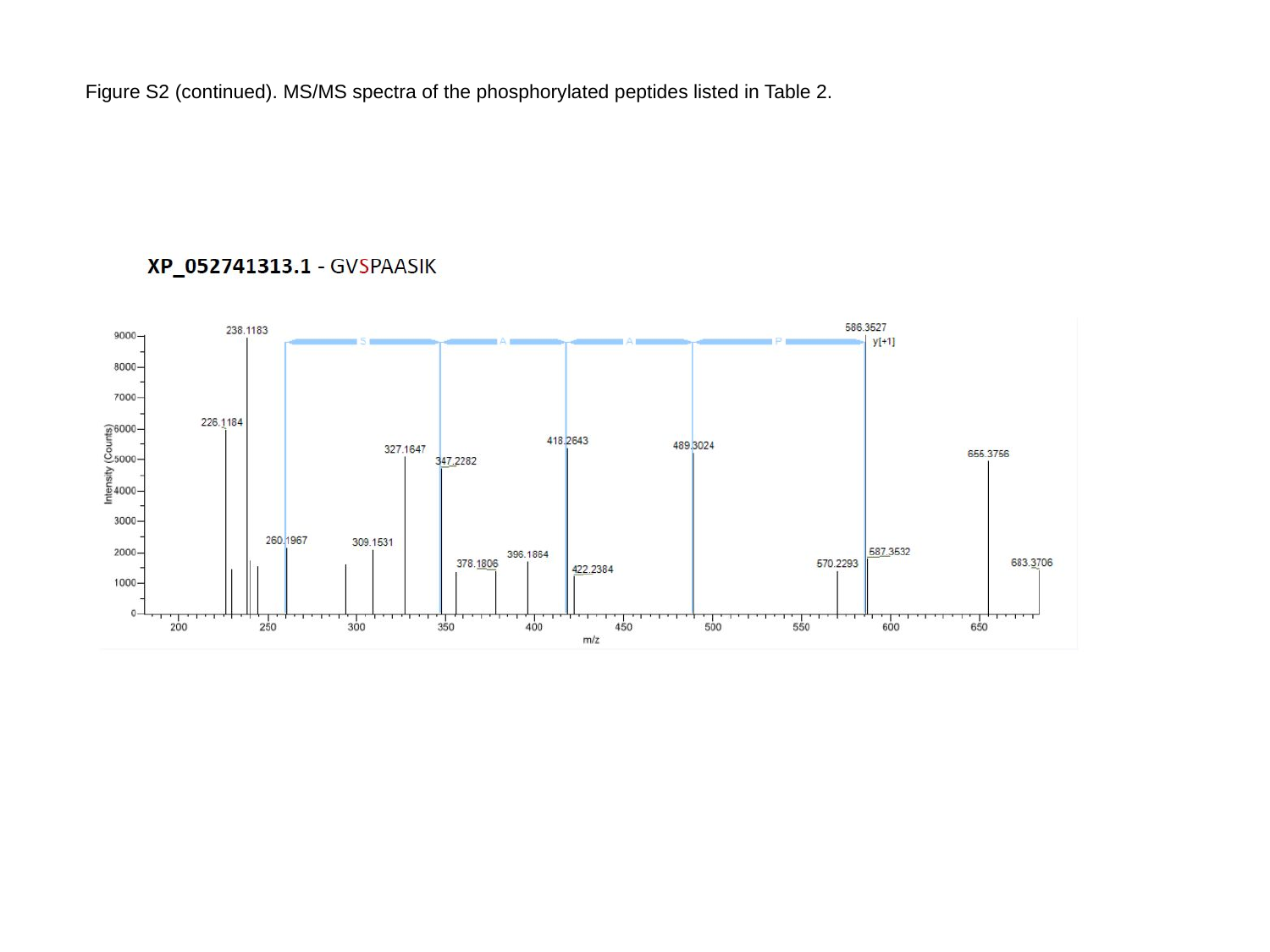

Figure S2 (continued). MS/MS spectra of the phosphorylated peptides listed in Table 2.

## Slide 8
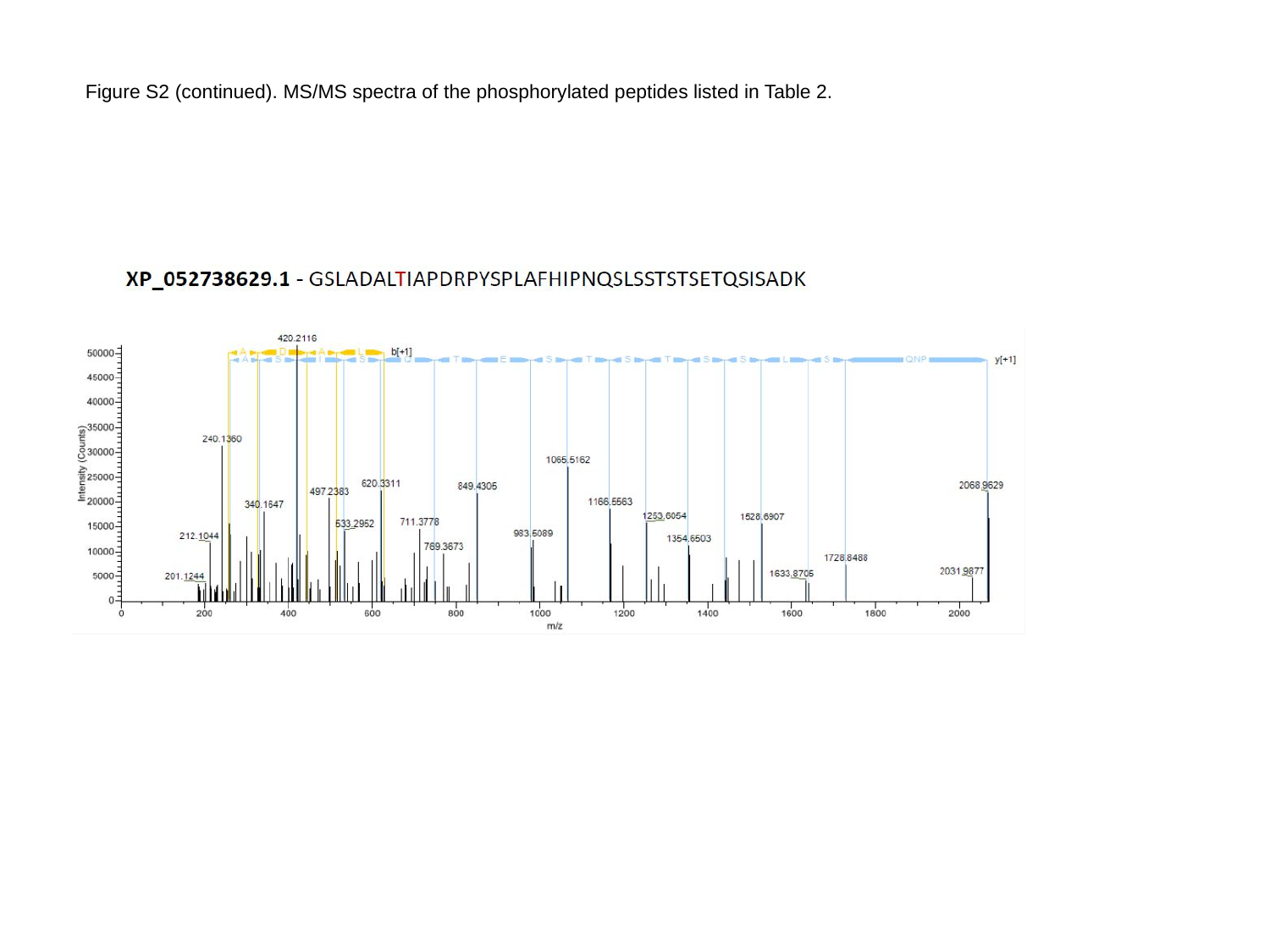

Figure S2 (continued). MS/MS spectra of the phosphorylated peptides listed in Table 2.

## Slide 9
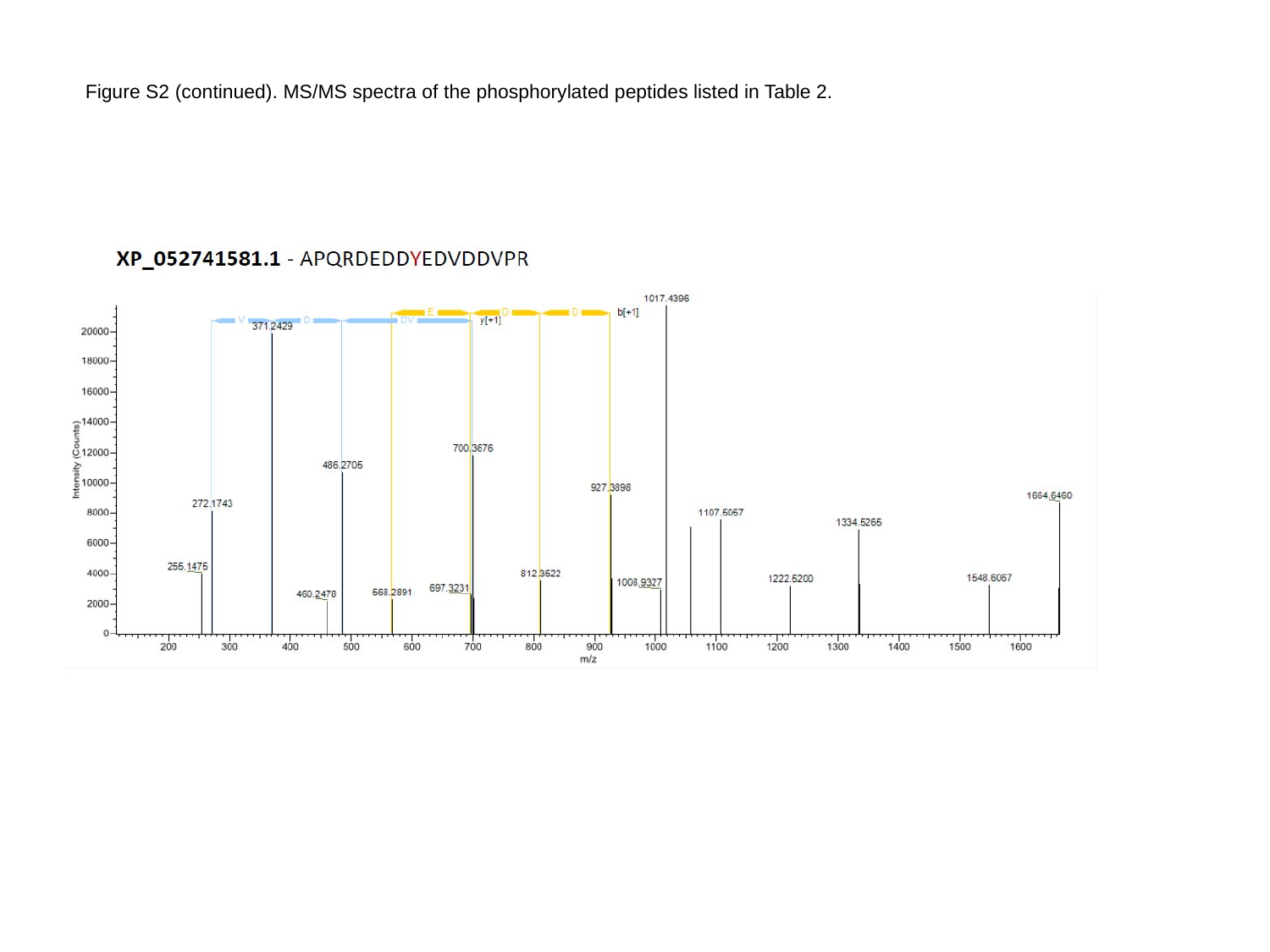

Figure S2 (continued). MS/MS spectra of the phosphorylated peptides listed in Table 2.

## Slide 10
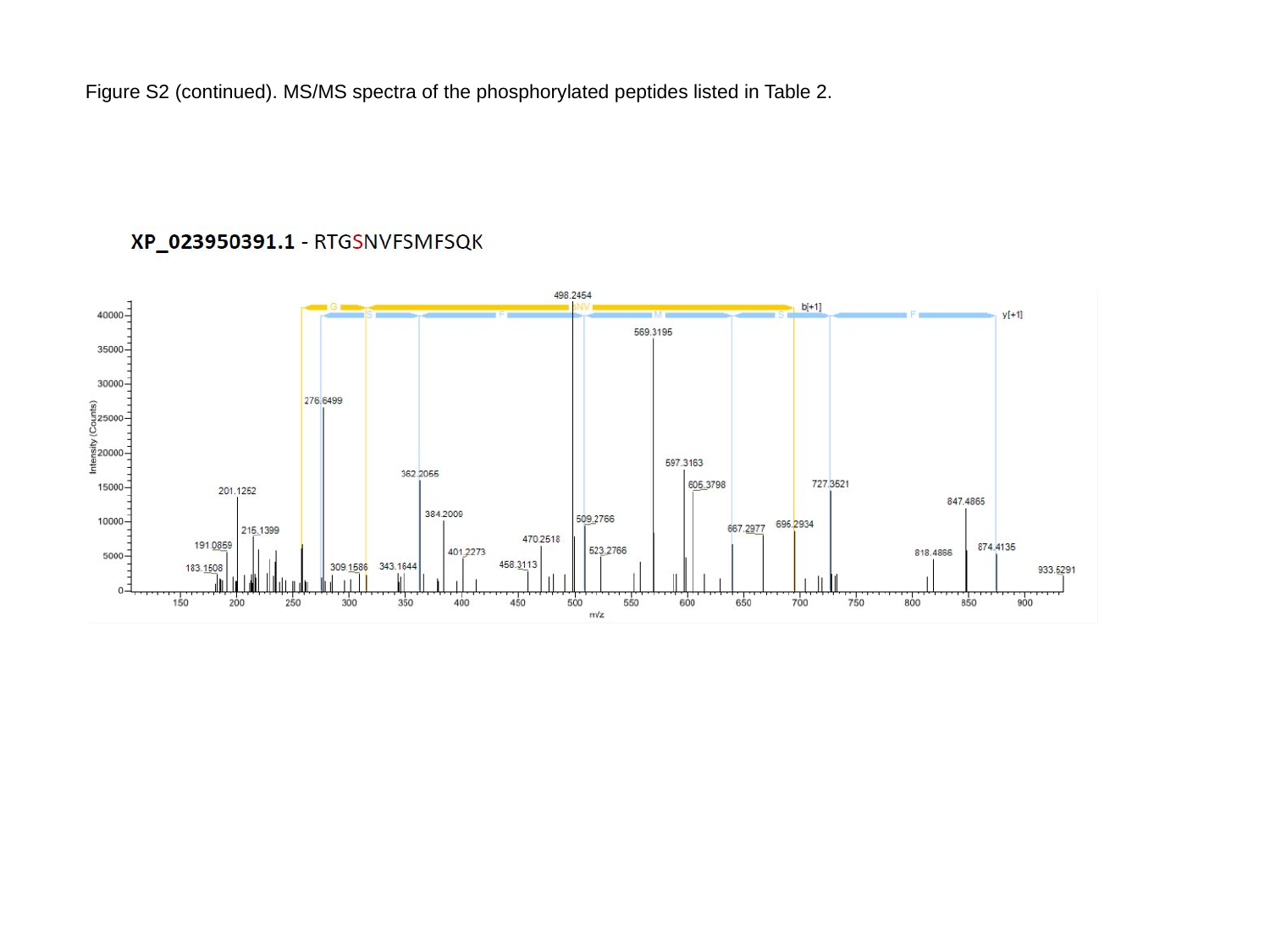

Figure S2 (continued). MS/MS spectra of the phosphorylated peptides listed in Table 2.

## Slide 11
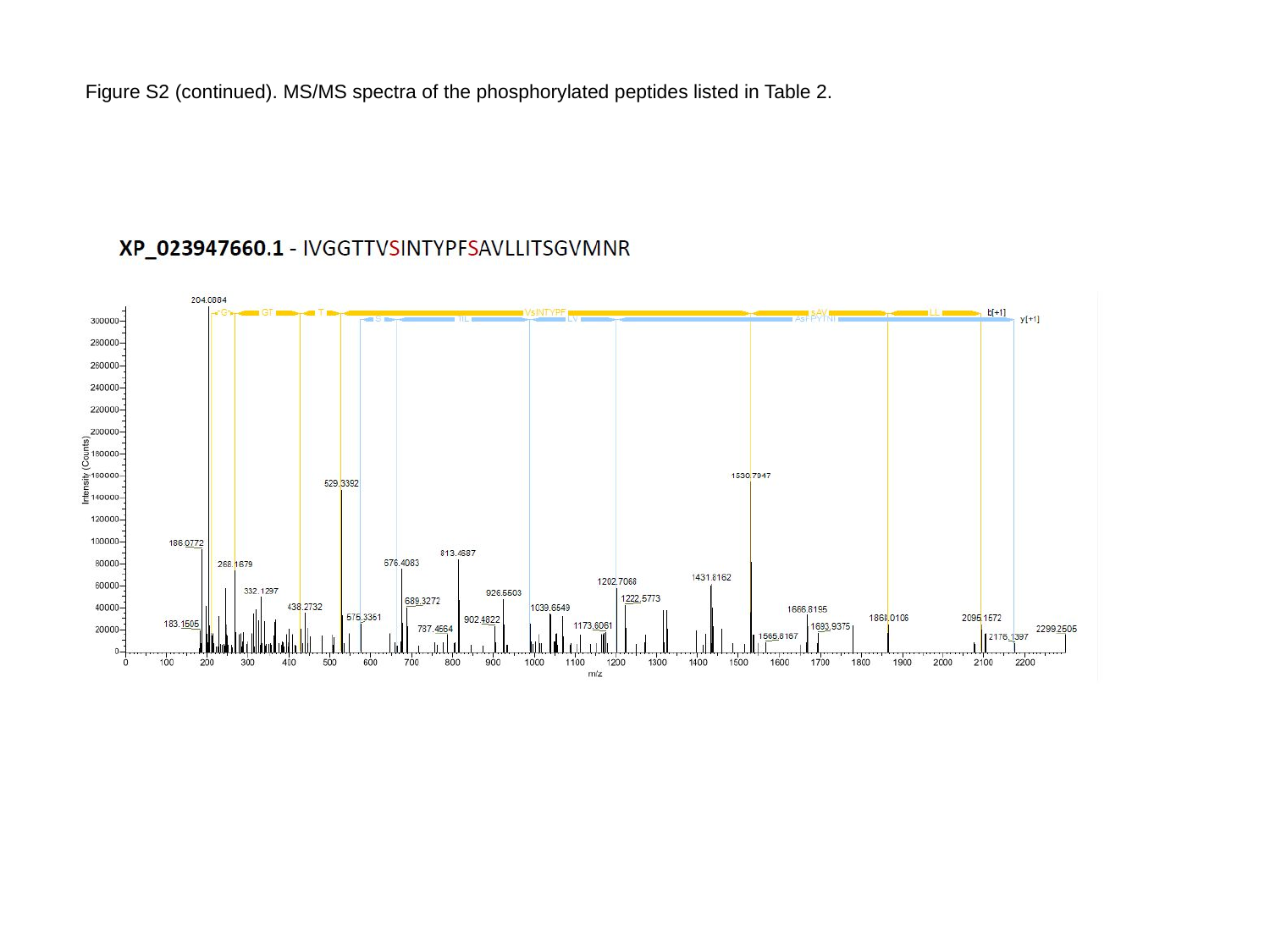

Figure S2 (continued). MS/MS spectra of the phosphorylated peptides listed in Table 2.

## Slide 12
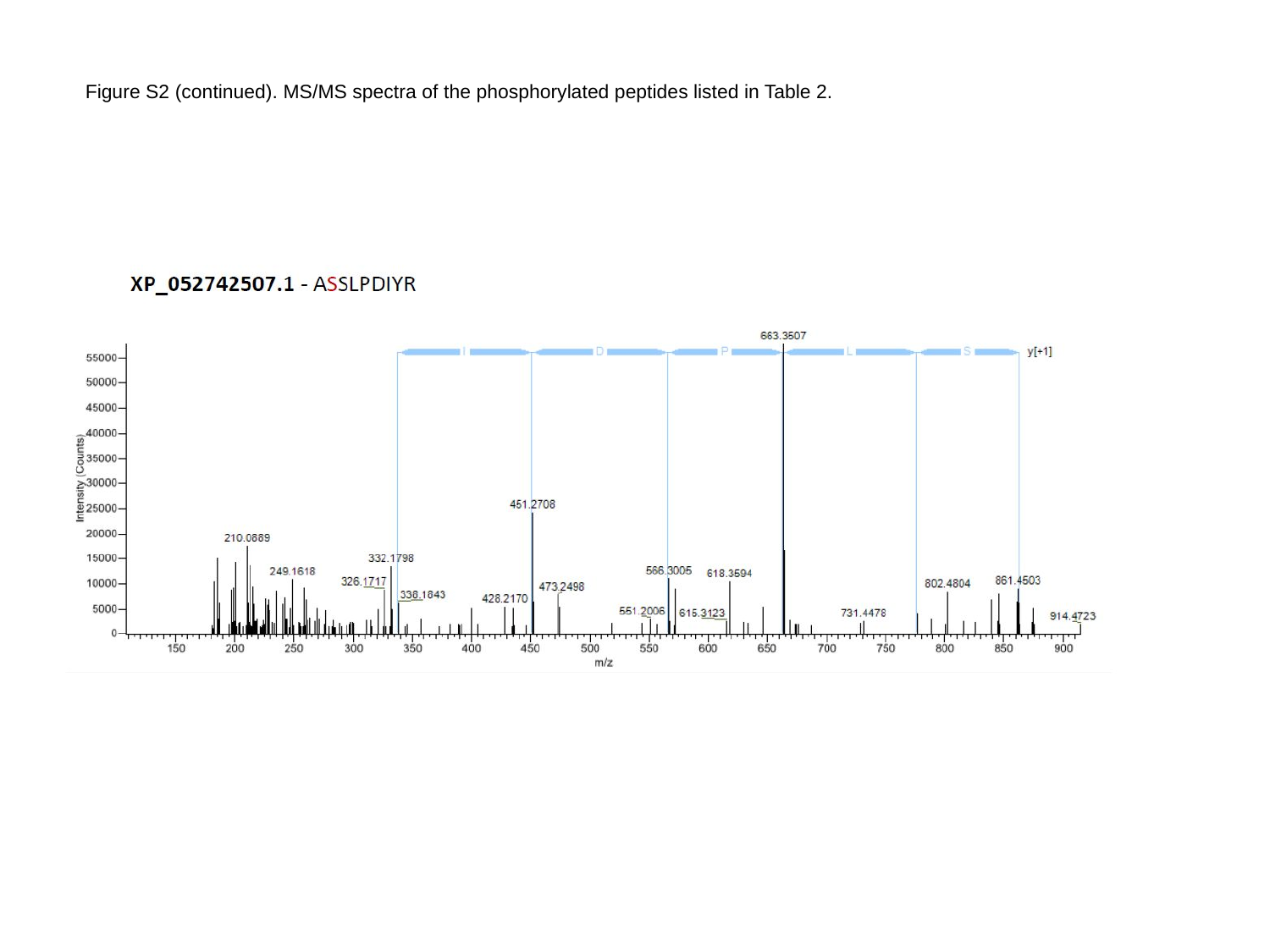

Figure S2 (continued). MS/MS spectra of the phosphorylated peptides listed in Table 2.

## Slide 13
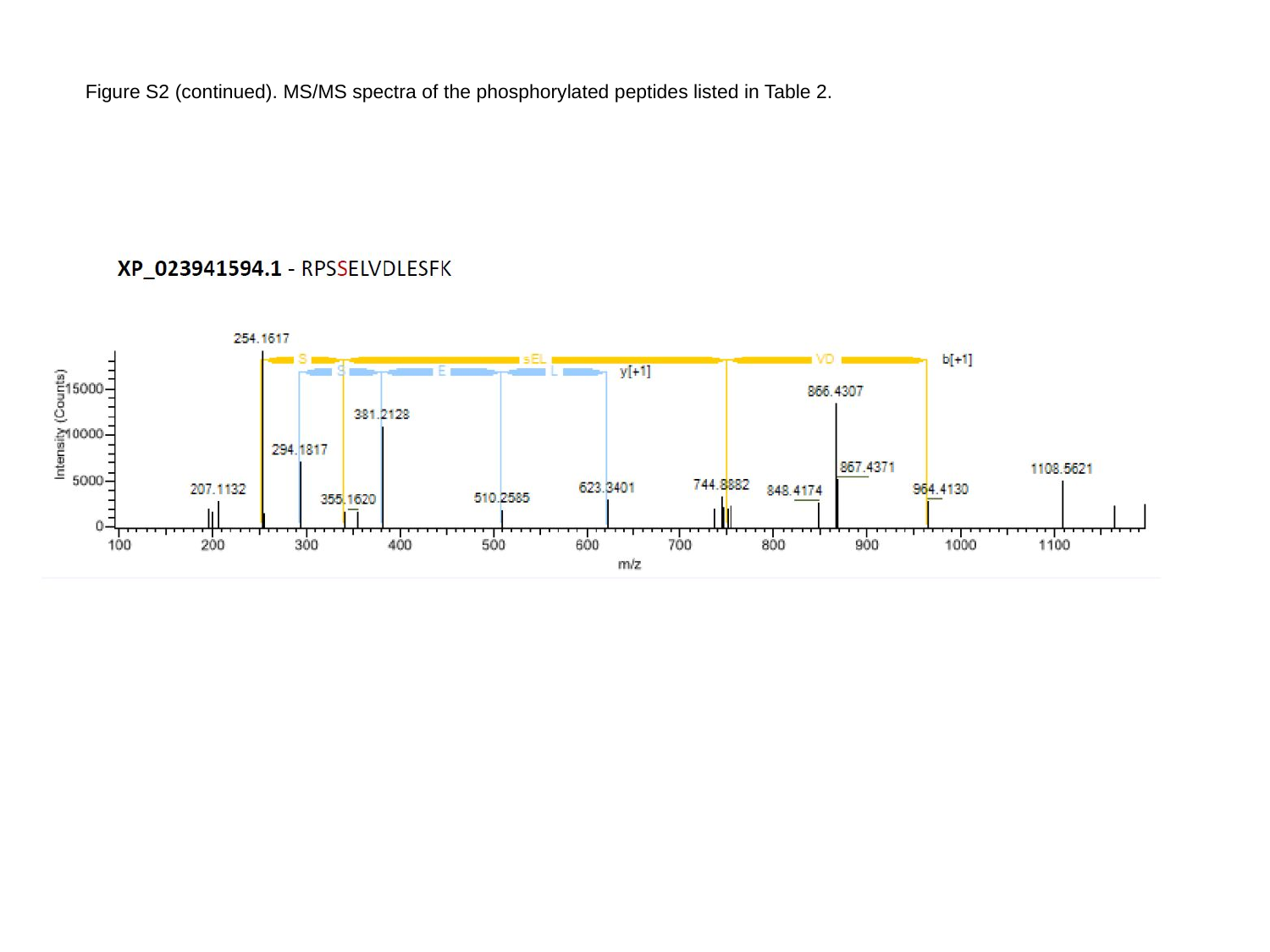

Figure S2 (continued). MS/MS spectra of the phosphorylated peptides listed in Table 2.

## Slide 14
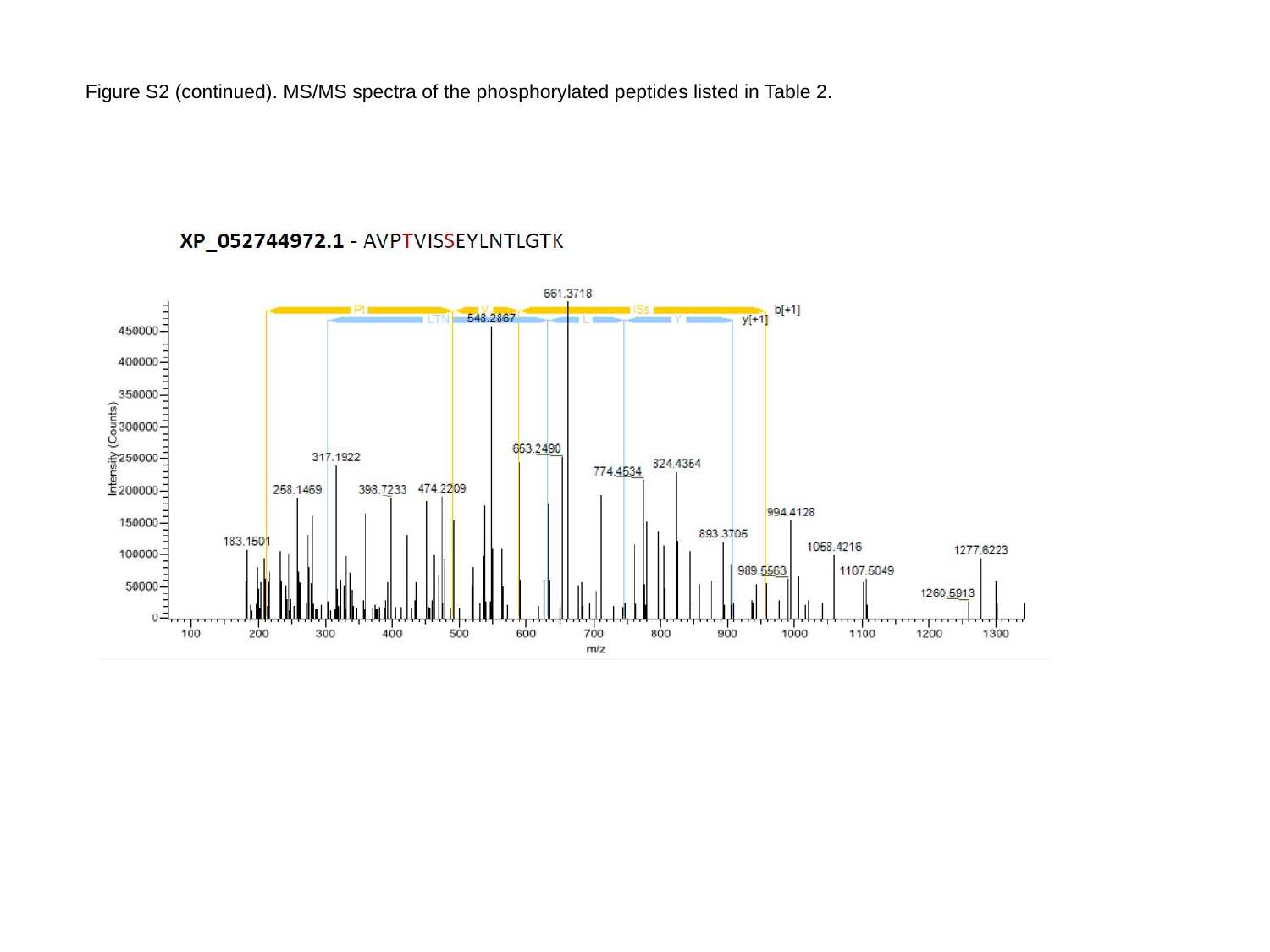

Figure S2 (continued). MS/MS spectra of the phosphorylated peptides listed in Table 2.

## Slide 15
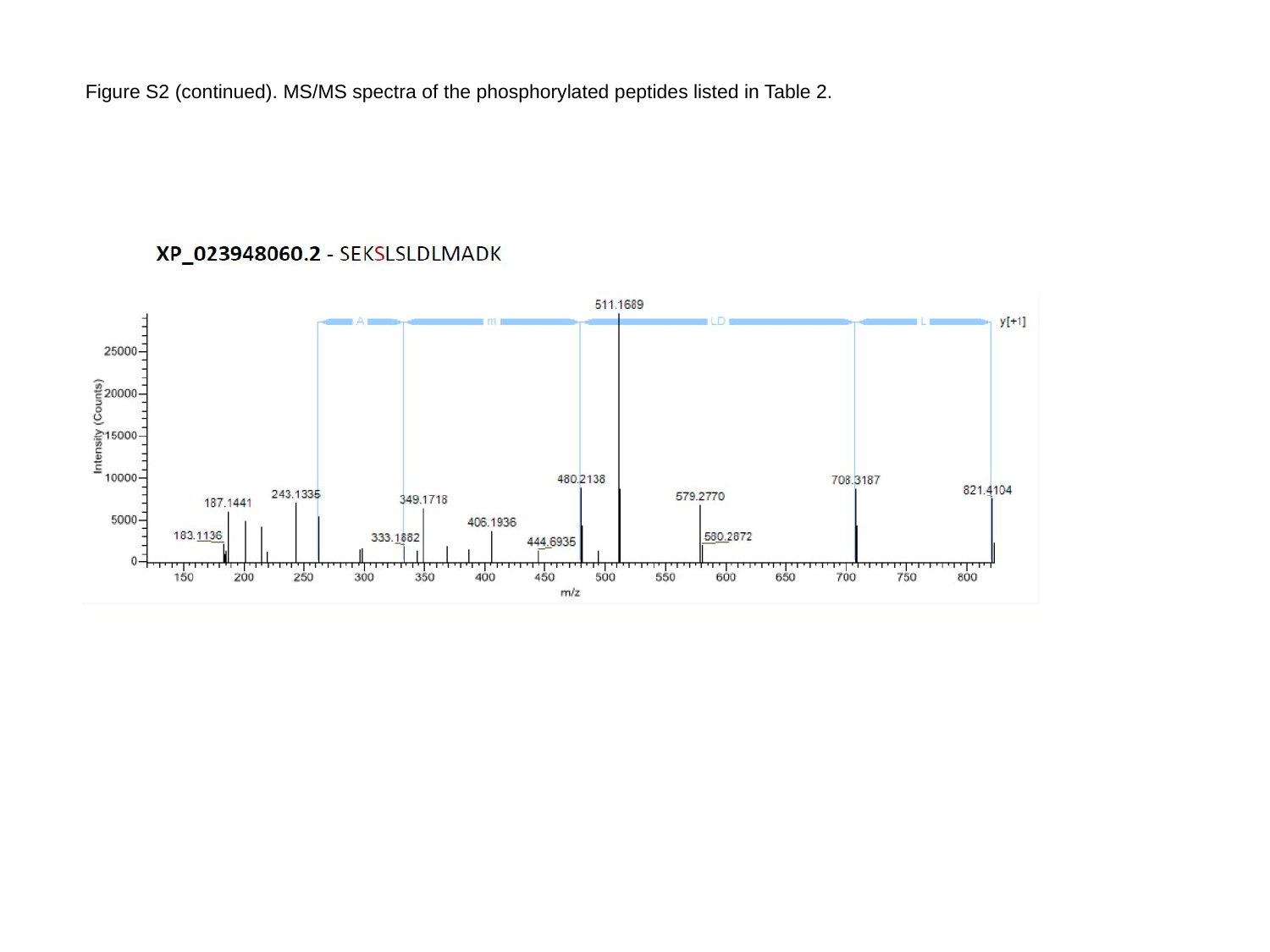

Figure S2 (continued). MS/MS spectra of the phosphorylated peptides listed in Table 2.

## Slide 16
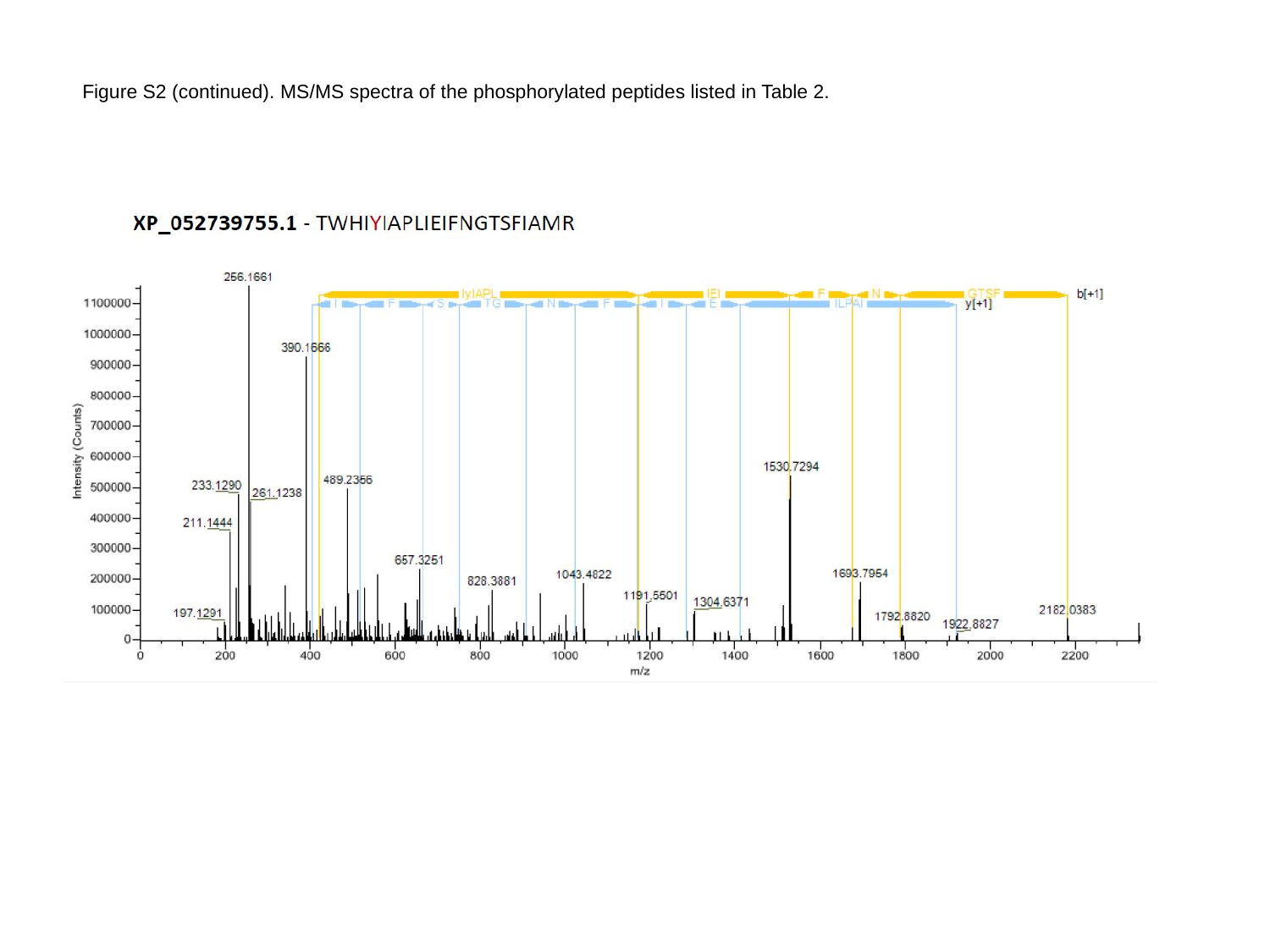

Figure S2 (continued). MS/MS spectra of the phosphorylated peptides listed in Table 2.

## Slide 17
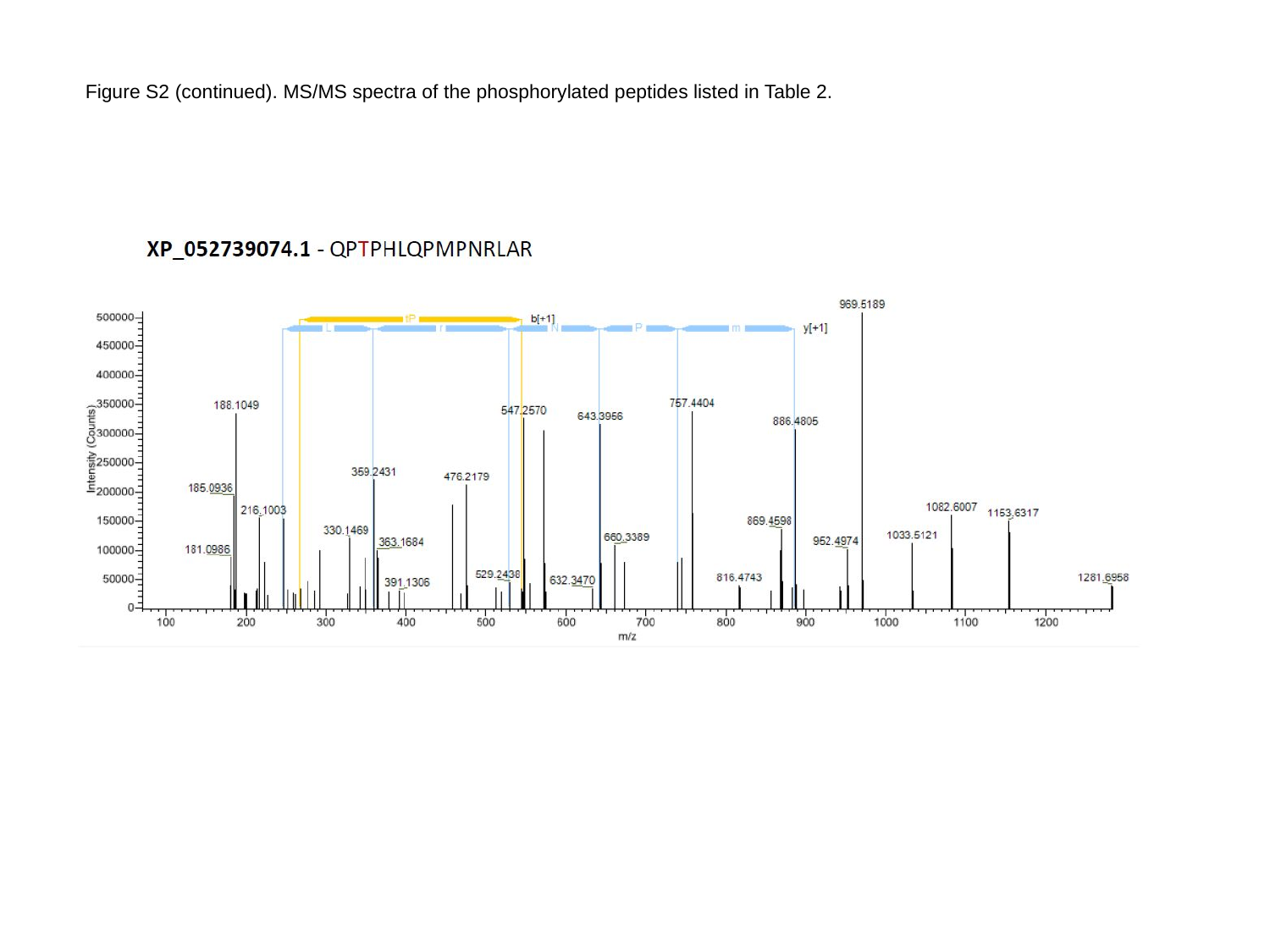

Figure S2 (continued). MS/MS spectra of the phosphorylated peptides listed in Table 2.

## Slide 18
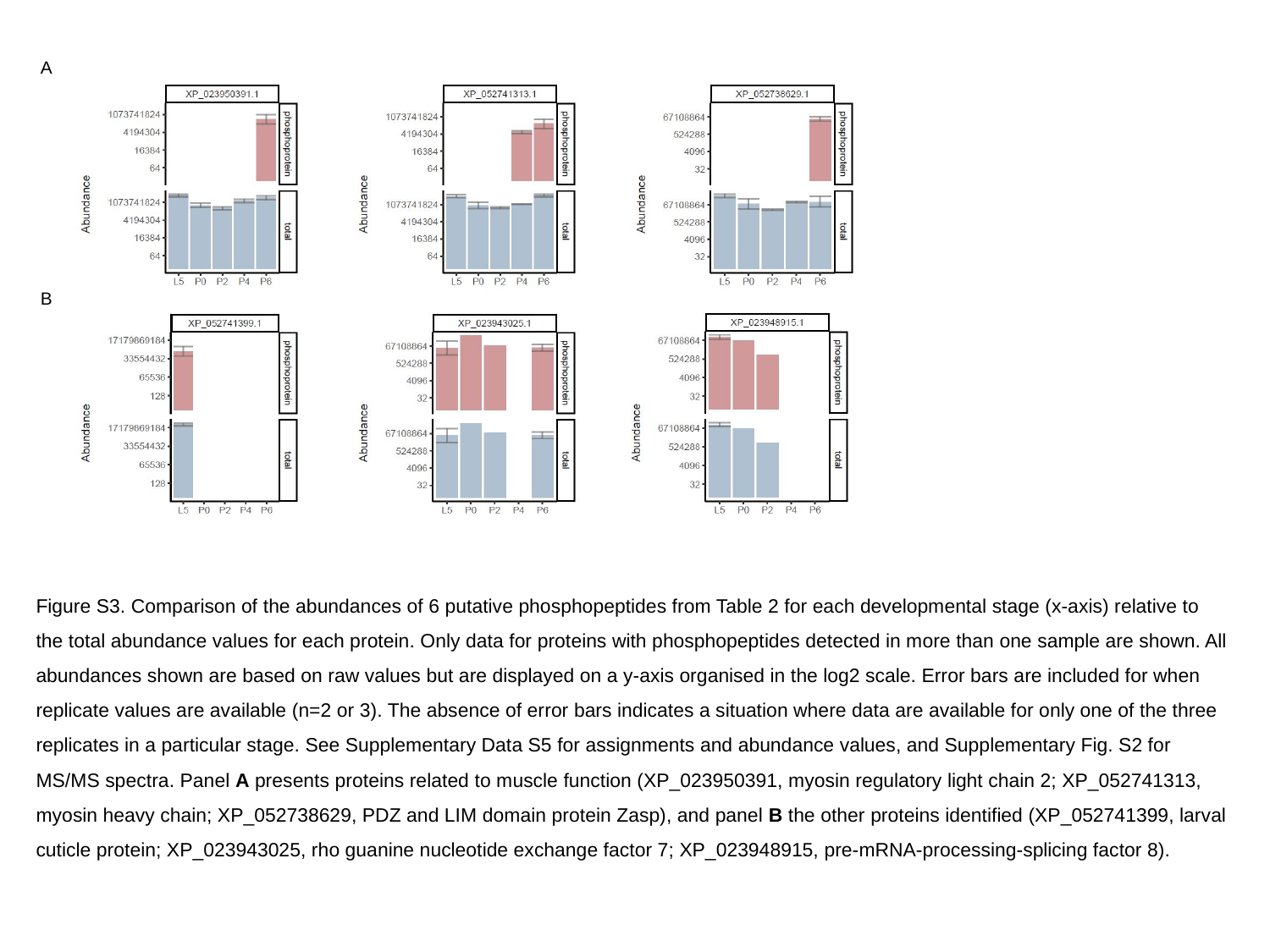

A
B
Figure S3. Comparison of the abundances of 6 putative phosphopeptides from Table 2 for each developmental stage (x-axis) relative to the total abundance values for each protein. Only data for proteins with phosphopeptides detected in more than one sample are shown. All abundances shown are based on raw values but are displayed on a y-axis organised in the log2 scale. Error bars are included for when replicate values are available (n=2 or 3). The absence of error bars indicates a situation where data are available for only one of the three replicates in a particular stage. See Supplementary Data S5 for assignments and abundance values, and Supplementary Fig. S2 for MS/MS spectra. Panel A presents proteins related to muscle function (XP_023950391, myosin regulatory light chain 2; XP_052741313, myosin heavy chain; XP_052738629, PDZ and LIM domain protein Zasp), and panel B the other proteins identified (XP_052741399, larval cuticle protein; XP_023943025, rho guanine nucleotide exchange factor 7; XP_023948915, pre-mRNA-processing-splicing factor 8).
